# Optimization Extraction, Preliminary Characterization and Antioxidant Activities of Polysaccharides from Semen Juglandis

**DOI:** 10.3390/molecules21101335

**Published:** 2016-10-09

**Authors:** Xueyong Ren, Liang He, Yanbin Wang, Junwen Cheng

**Affiliations:** 1 College of Materials Science and Technology, Beijing Forestry University, Beijing 100083, China; 2 Key Laboratory of Biological and Chemical Utilization of Zhejiang Forest Resources, Department of Forest Foods, Zhejiang Forestry Academy, Hangzhou 310023, China; kite006@163.com (L.H.); yanbinwangzjfa@163.com (Y.W.); chengjunwen2014@163.com (J.C.)

**Keywords:** Semen Juglandis, polysaccharide, response surface methodology (RSM), physicochemical property, antioxidant activities

## Abstract

The optimization extraction process, preliminary characterization and antioxidant activities of polysaccharides from Semen Juglandis (SJP) were studied in this paper. Based on the Box-Behnken experimental design and response surface methodology, the optimal extraction conditions for the SJP extraction were obtained as follows: temperature 88 °C, extraction time 125 min and ratio of liquid to solid 31 mL/g. Under these conditions, experimental extraction yield of SJP was (5.73 ± 0.014)% (*n* = 5), similar to the predicted value of 5.78%. Furtherly, the purified SJP obtained from SJP extract by DEAE-52 and Sephacryl S-100 chromatography was analyzed to be rhamnose, galacturonic acid, galactose, arabinose and fucose in the molar ratio of 1:6.34:1.38:3.21:1.56. And the weight-average molecular weight and radius of gyration of the purified SJP in 0.1 M NaCl were determined to be 2.76 × 10^4^ g/mol and 122 nm by SEC-MALLS, respectively. More importantly, it exhibited appreciable antioxidant activities compared to the standard Vc, such as DPPH radical scavenging activity (IC_50_ 0.21 mg/mL), strong reducing power, ABTS radical scavenging activity (IC_50_ 0.29 mg/mL), and hydroxyl radical scavenging activity (IC_50_ 0.38 mg/mL). These results indicate that SJP may be useful for developing functional health products or natural antioxidant.

## 1. Introduction

Walnut (*Juglans regia* L.), belonging to family Juglandaceae, is originated in south-eastern Europe, Asia Minor, India, and China [1]. This kind of plant is not only a valuable agricultural goods, but its leaves, barks, stems, pericarps, fruits, flowers, and ligneous membranes are all extensively applied for various medicinal therapy in China. Semen Juglandis, treated as byproducts of walnut production, was normally discarded or burned directly as fuel in the past [2]. Some researchers have focused on the biological activities of their aqueous extracts and ethanol extracts from the raw materials of Semen Juglandis and the results indicated that this drug possessed significant anti-deficiency of kidney YANG and anti-inflammatory effects [3,4]. Referring to literature, some low-molecular-weight, soluble compounds of Semen Juglandis may attribute to its related biological activities [5,6], such as total alkaloids, flavonoids, saponins, pheonols or other components.

A large body of evidence suggests that oxidative damage caused by free radicals is related to aging and diseases, such as atherosclerosis, cancer and rheumatoid arthritis. Some macromolecules have attracted increasing attention in using as antioxidants for dietary supplements with the function of protecting the human body from the oxidative damage. Polysaccharide existed in many natural plants or microorganism, has been reported to possess pharmacological properties including antioxidant, antibacterial, antitumor activities and immunostimulation [7,8,9,10,11]. On the other hand, most of bioactive polysaccharides have been found to be closely related with their various chain conformations in solution, such as the molecular weight and chain conformation [12]. Some valuable information on the molecular characteristics of the polymer, such as z-average root-mean square radius of gyration (*R*_g_^2^)_z_^1/2^, weight-average molecular weight and polydispersity index, could provide insights into its physico-chemical behavior and indicate effective application of the biopolymer [13]. Therefore, it is essential to acquire basic parameters of biomacromolecules in solution for the development of health products as antioxidants. However, the research on extraction and evaluation of new polysaccharides from Semen Juglandis is still scare, which is in urgent need for the relevant product exploitation.

In the case of extraction process, a great variety of factors, i.e., extraction temperature, extraction time and solvent to solid ratio mainly affect yields of bioactive compounds from valuable natural resources. Response surface methodology (RSM) is a collection of mathematical and statistical techniques extensively utilized to optimize the extraction process in many fields [14,15]. Box-Behnken design (BBD) of RSM, having only three levels and fewer experiments involved, is less laborious and time-consuming than other approaches to arrange experiments, evaluate the model and determine multiple factors as well as possible interactions between independent variables [16,17].

The objective of this study was employed to optimize the extraction process parameters of water-soluble polysaccharide from Semen Juglandis (SJP) using Box-Behnken design, followed by canonical and ridge analyses. After the fractionation of the crude SJP by DEAE-52 and Sephacryl S-100 column, the physicochemical properties of main fraction were carried out by HPLC and SEC-MALLS analysis as well. Moreover, the antioxidant scavenging effects of the fraction were evaluated by in vitro antioxidant assay, including tests of DPPH radical scavenging activity, reducing power, ABTS radical scavenging activity and hydroxyl radical scavenging activity.

## 2. Results and Discussion

### 2.1. Optimization of the SJP Yield

On the basis of single factor experimental results, the 3 independent variables of extraction temperature, extraction time and ratio of solution to solid were analyzed to optimize the SJP yield from Semen Juglandis. A total of 17 experimental runs were performed with different combinations of these 3 factors. Experimental and predicted values were shown in Table 1. There was a considerable variation in the SJP yield depending on different extraction conditions.

Using multiple regression, data obtained were analyzed based on Equation (1) with the help of Design Expert software version 7.1.3. The predicted response of *Y* for SJP was as follows:

*Y*% = 5.67 + 0.39*X*_1_ + 0.35*X*_2_ + 0.21*X*_3_ − 0.41*X*_1_*X*_2_ − 0.03*X*_1_*X*_3_ − 0.37*X*_2_*X*_3_ − 0.48*X*_12_ − 0.48*X*_22_ − 0.49*X*_32_ (1)

where *Y* is the predicted response variable (the yield of SJP as a %), and *X*_1_, *X*_2_, and *X*_3_ are coded values of the independent variables of extraction temperature, extraction time, and ratio of solution to solid, respectively.

The statistical significance of Equation (1) was checked using Fisher’s statistical ANOVA test (*F*-test) and results are shown in Table 2. The *F*-test indicated that the second model had a high model *F* value of 17.46 and a low *p* value of *p* < 0.0005, indicating the model was suited well for the experimental data. The adjusted coefficient of determination (*R*^2^*_adj_*) indicated that the sample variation of 90.25% for the SJP yield was attributable to the independent variables. The value of *R*^2^ (0.9573) showed that the experimental results fit well with the theoretical values predicted by the polynomial model [18]. A relatively low value of C.V.% (4.26%) illustrated further the experiments were practical with a better precision and reliability. The coefficient estimates of Equation (1), along with the corresponding *p*-values are presented in Table 2 as well. It can be seen that most of regression coefficients are significant except for two linear coefficients (extraction temperature *X*_1_ and ratio of solution to solid *X*_3_).

### 2.2. Analysis of Response Surface

**Figure 1:**
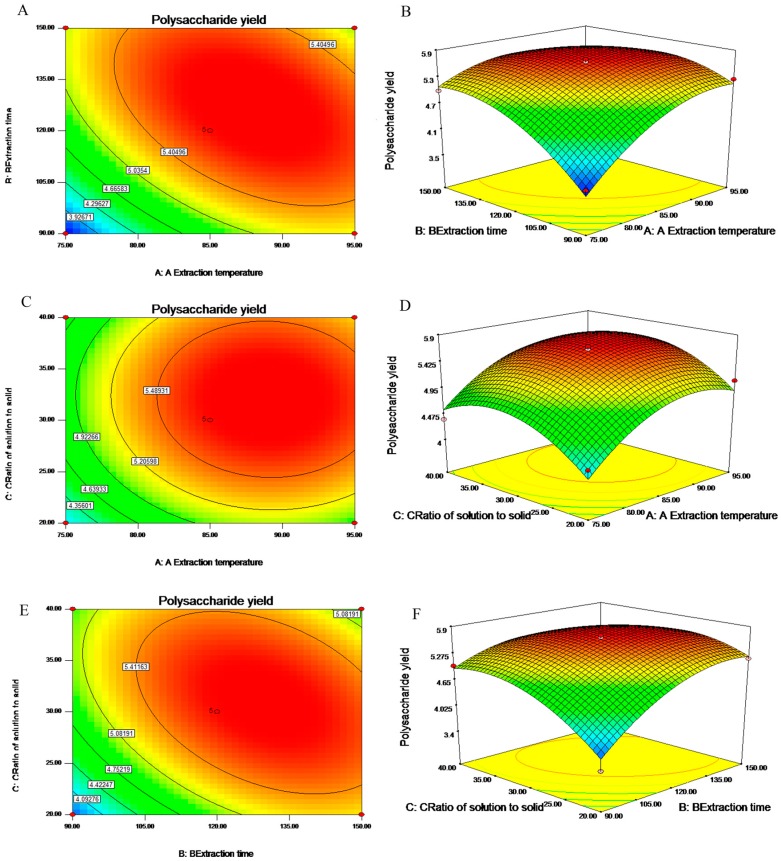
Response surface (**left**) and contour (**right**) plots of the effects of 3 variables, extraction temperature (*X*_1_), extraction time (*X*_2_) and ratio of solution to solid (*X*_3_) on the yield of SJP extraction: (**A**) response surface showing the effect of extraction temperature and extraction time on the yield of SJP extraction; (**B**) contour plot showing the effect of extraction temperature and extraction time on the yield of SJP extraction; (**C**) response surface showing response surface showing the effect of extraction temperature and ratio of solution to solid on the yield of SJP extraction; (**D**) contour plot showing the effect of extraction temperature and ratio of solution to solid on the yield of SJP extraction; (**E**) response surface showing the effect of extraction time and ratio of solution to solid on the yield of SJP extraction; (**F**) contour plot showing the effect of extraction time and ratio of solution to solid on the yield of SJP extraction.

The fitted response surface plots and their corresponding contour plots for SJP extraction by the previous model were shown in Figure 1, in which it provides a method to visualize the relation between the response and experimental levels of each variable, and the type of interaction among the testing variables with an aim of optimum conditions.

Figure 1A,B depicted the 3D-plot and its respective contour plot showing the effects of *X*_1_ (extraction temperature) and *X*_2_ (extraction time) on the yield of SJP, while *X*_3_ (ratio of solution to solid) was fixed at middle value of 30 mL/g (Actual). An elliptical shape of the contour plot indicated that an interaction between *X*_1_ and *X*_2_ was found to contribute to the yield of SJP at a significant level. This fact could be well accounted for by *p*-value (*p* < 0.0062) in Table 2. From Figure 1B, it was evident that when the extraction temperature was from 75 to 88 °C, and the extraction time was from 90 to 125 min, the SJP yield was over 5.78% and then decreased slowly beyond this range. These facts were important in making the whole extraction process economically more feasible and made it possible to save energy in future industrial application. Those were consistent with the results of *Bletilla striata* polysaccharides [19]. They reported that an increase in the yield of polysaccharides could be significantly achieved with the increases of extraction temperature but would not increase any longer. The reason could probably be explained that the increasing energy can promote the process of extraction when the temperature and extraction time are at relatively low value.

There was no significant interaction relationship between *X*_1_ (extraction temperature) and *X*_3_ (ration of solution to solid) existing in Figure 1C, which could be proved by *p*-value (0.7886) in Table 3. It was obvious that the yield of SJP climbed up continuously when the value of *X*_1_ increased from 75 to 88 °C. Then it went down slightly outside that optimum point. The curve of ratio of solution to solid in the contour plot was slightly inclined to horizontal showing that the yield of SJP was up to a saturated value regardless of the increasing *X*_3_. With the assistance of ratio of solution to solid on the initial process of SJP from Semen Juglandis, it enhanced the diffusion process and provided a greater space for components into solvent during the extraction. However it had no effect on the yield of SJP at relatively high ratio of solution to solid, which might be explained by decreasing the available surface area between water solvent and the cells. Those findings could also be found in the report made by Sun et al. [20].

The 3-D response surface plot and the contour plot based on independent variables extraction time and ratio of solution to solid were shown in Figure 1E,F. The circinal nature of the contour plots indicated that no interactions between *X*_2_ (extraction time) and *X*_3_ (ratio of solution to solid) were found to contribute to the response at a significant level. This fact also could be accounted for by *p*-value (0.0101) in Table 2. It could be seen from Figure 1F that SJP yield increased gradually with the increasing extraction time and ratio of solution to solid. While the extraction time was at low level, the effect of extraction temperature on the response was insignificant. It was obvious that the yield of SJP was higher than 5.78% when extraction time was in the range of 90 to 125 min (actual value) and ratio of solution to solid in the range of 20 mL/g to 31 mL/g. Yin and Dan [21] reported that water treatment played a critical role in the extraction of polysaccharides by easily hydrolyzing the *O*-glycosidic bonds.

### 2.3. Optimization of Extracting Parameters and Validation of the Model

By applying the software of Design-Expert (Version 7.1.3), the maximum predicted value and the predicted optimum conditions for SJP extraction could be obtained quietly. Those were as following: extraction temperature 88.28 °C, extraction time 125.25 min and ratio of solid to liquid 31.40 mL/g, and a maximum response of 5.78% was predicted by the model equation. However, considering operation under actual production conditions, the optimal conditions can be modified as follows: extraction temperature 88 °C, extraction time 125 min and ratio of solid to liquid 31 mL/g. In order to confirm the predicted value was not bias toward the practical result, further experiments were carried out under the slightly modified optimal conditions. A mean value of (5.73 ± 0.014)% (*n* = 5) acquired from real experiments, which validated the reasonableness of the RSM model. It drew a conclusion that the model of Equation (1) developed was considered to be satisfactory and accurate for predicting the extraction yield of SJP.

### 2.4. Preliminary Characterization of the Purified SJP

**Figure 2:**
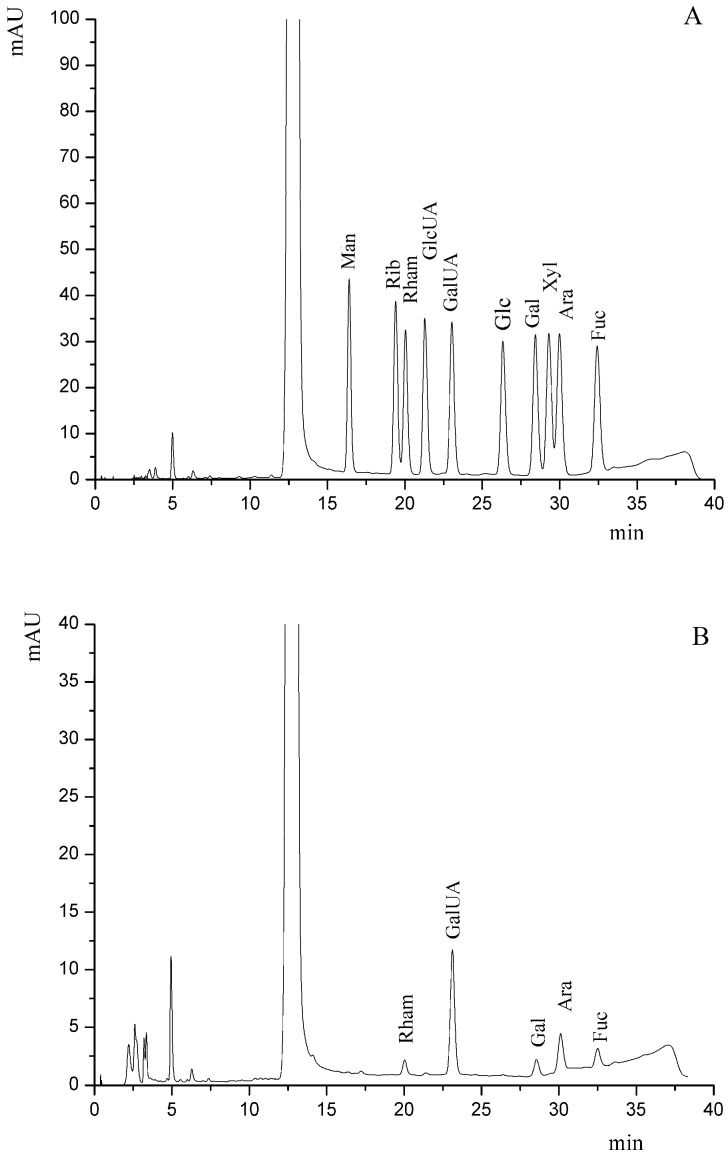
HPLC chromatograms of PMP derivatives of component monosaccharides released from (**A**) sugar standards(Peaks from left to right: Mannose, Ribose, Rhamnose, Glucuronic acid, Galacturonic acid, Glucose, Galactose, Xylose, Arabinose, Fucose with retention time from 15 to 35 min) and (**B**) the purified SJP.

The composition of the purified SJP shown in Figure 2, determined by HPLC analysis as PMP derivatives, indicated that it was composed of rhamnose, galacturonic acid, galactose, arabinose and fucose in the molar ratio of 1:6.34:1.38:3.21:1.56. In term of peak area, the two predominant monosaccharides was galacturonic acid, which was similar with the results of non-starch polysaccharide from American ginseng by Guo et al. [22] and they reported that the dominate component in GSP was galacturonic acid up to 70% in molar ratio. The detection of Rham/GalUA residues implied that the purified SJP might be RG-I type heteropolymers with a backbone of 1,4-linked-α-galacturonic acid residues due to the small ratio (0.15) of Rham/GalUA [23].

**Figure 3:**
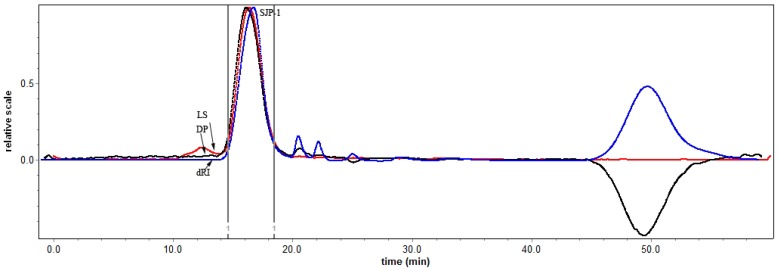
Elution profile of the purified SJP in a SEC-MALLS system.

The SEC-MALLS approach can provide great insight into the characterization of biomacromolecules by automatically measuring the various physical properties of sample including molar mass, hydrodynamic sizes and distribution. And the advanced conformation of biopolymers has been reported to be closely related to their biological activities [24]. Figure 3 showed the elution profile of the purified SJP for the determination of molecular mass, in which it presented a single peak monitored both by both LLS and RI detectors. After the calculation of its molar mass with the formula of Rayleigh-Debyte, the number-average molecular weight (*M*_n_), weight-average molecular weight (*M*_w_) and z-average molecular weights (*M*_z_) of the purified SJP in 0.1 mg/mL NaCl were determined to be 2.81 × 10^4^ g/mol, 2.76 × 10^4^ g/mol and 2.94 × 10^4^ g/mol, respectively. Interestingly, Wang et al. [24] reported that the triple-helix glucan with *M*_w_ of 5.1 × 10^6^ g/mol was obtained from water-soluble polysaccharides in *Dictyophora indusiata*, which was quite similar with that of water-soluble polysaccharide. The (*R*_g_^2^)_z_^1/2^ value, one of parameters of macromolecules, was usually a measure regarding how far from the center of mass and how the mass of the polymer chains was concentrated. The bioactive glucan had been found to have a potent antitumor activity due to its advanced conformation with an (*R*_g_^2^)_z_^1/2^ of 141 nm. A (*R*_g_^2^)_z_^1/2^ value of 122 nm for the purified SJP reflecting its polymer chains with more compact conformation showed that it might have a similar antitumor activity with glucan. Furthermore, the 1.038 of polydispersity (*M*_w_/*M*_n_) indicated that the purified SJP has a relatively narrow molecular-weight distribution.

### 2.5. Antioxidant Activities of SJP

#### 2.5.1. DPPH Scavenging Activity

**Figure 4:**
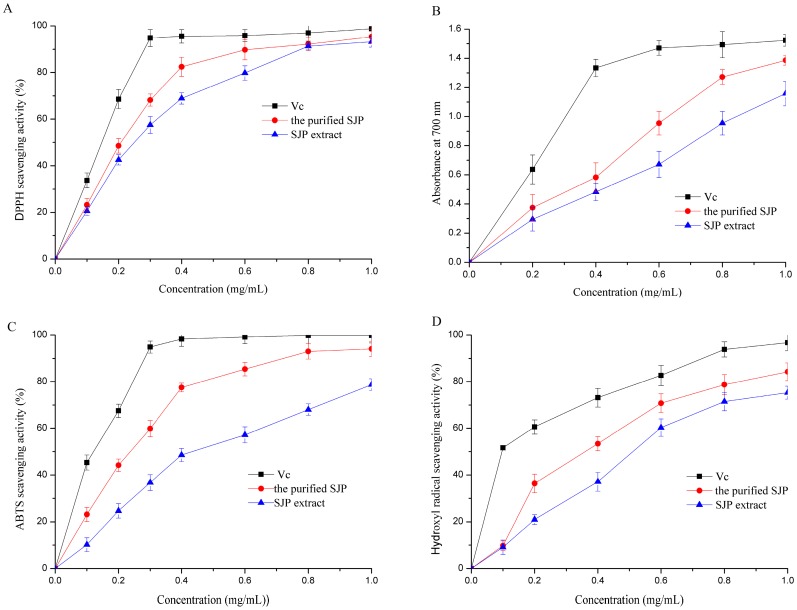
Antioxidant activities of SJP extract, the purified SJP and Vc at different concentrations: (**A**) DPPH radical scavenging activities; (**B**) reducing power activities; (**C**) ABTS radical scavenging activities and (**D**) hydroxyl radical scavenging activities. Each data are presented as the mean value of triplicate measurements.

The result about DPPH scavenging activities of reference standard ascorbic acid, SJP extract and the purified SJP obtained from Semen Juglandis were presented in Figure 4A. On the whole, the scavenging rates of SJP extract and the purified SJP from Semen Juglandis were quite similar with standard Vc within the test dosage range, which suggested that both SJP extract and its purified had obvious effects on scavenging free DPPH radical. Furthermore, their scavenging activities increased very significantly with increasing concentrations. At 0.1 mg/mL, the scavenging values of two testing samples were above 15%, higher than that of aqueous extract of walnut husks (3.81%) [25]. Increasing from dose of 0.2 to dose of 0.8 mg/mL, the purified SJP presented a noticeable hydrogen would be donated to combine with DPPH radical when SJP extract was purified furtherly on a relatively low concentration. And the IC_50_ value of SJP extract and the purified SJP were found to be 0.26 mg/mL and 0.21 mg/mL, respectively. This means that SJP from Semen Juglandis could be accepted as primary antioxidants.

#### 2.5.2. Reducing Power Activity

Reducing power measures the reductive ability, which is evaluated by the transformation of Fe(III) to Fe(II) in the presence of antioxidants. The results of reductive capability about two samples from Semen Juglandis were demonstrated in Figure 4B, and compared to ascorbic acid which plays the role of standard. The measurement of reducing power of SJP extract and the purified SJP determined at 700 nm showed dose-dependent response. As it can be seen in those results, the absorbance value of ascorbic acid in the range of measured concentration was slightly higher than those of two testing ones. Moreover, the scavenging ability of the purified SJP was more active than SJP extract at relative low concentration of 0.1 to 0.8 mg/mL. And then both of the reducing powers reached a plateau of 1.16–1.38. These data suggest that SJP could be an effective electron donor capable of reacting with free radicals to convert them into more stable products.

#### 2.5.3. ABTS Scavenging Activity

The ABTS^+^ assay can be used as an index to reflect the antioxidant activity of the test samples [26]. The scavenging ability of SJP extract and the purified SJP on ABTS free radicals increased obviously (*p* < 0.05) with the increasing concentration presented in Figure 4C. The IC_50_ values of SJP extract and the purified SJP were 0.43 mg/mL and 0.29 mg/mL, respectively. As illustrated in the figure, the purified polysaccharide possessed stronger ABTS scavenging ability than SJP extract. Furtherly, at higher concentration (0.8 mg/mL), the activity of the purified one was almost equivalent to ascorbic acid but lower than the later.

#### 2.5.4. Hydroxyl Radical Scavenging Activity

As for hydroxyl radical scavenging activity, there are two types of reaction mechanism: one suppresses the generation of the hydroxyl radical, and the other removes the hydroxyl radicals generated [27]. This method was carried out using Fenton’s reaction. As described in Figure 4D, the hydroxyl radical-scavenging effect of SJP extract and the purified SJP, in a concentration of 0.6 mg/mL, was found to be 60.32% and 70.78% and. Ascorbic acid, which was used as a standard since it is reported to be significantly (*p* < 0.05) effective in inhibition of hydroxyl radicals, showed 82.72% scavenging effect at a concentration of 0.6 mg/mL. The IC_50_ values were tested to be 0.52 mg/mL and 0.38 mg/mL for SJP extract and the purified SJP. It was obvious that the scavenging effect of the purified polymer was relatively higher than that of SJP extract from the range of 0.2 mg/mL to 1.0 mg/mL. Yang et al. [28] also reported that the major fraction showed higher antioxidant than crude polysaccharide from Ficus carica L, which was in a good agreement with the findings as expressed here.

## 3. Materials and Methods

### 3.1. Materials and Chemicals

The raw walnuts with Semen Juglandis were collected from Kunming, Yunnan Province, China. Monosaccharide standards were purchased from Sigma-Aldrich Chemical Co. (St. Louis, MO, USA). Bovine serum albumin (BSA) was purchased from Sijiqing Biological Engineering Materials Co. (Hangzhou, China). DEAE-52 column was purchased from Whatman Co. (Maidstone, Britain). Sephacryl S-100 and T-series Dextrans were purchased from Pharmacia Co. (Sweden). Glucose, peptone, ethanol, yeast extract, KH_2_PO_4_, MgSO_4_, FeSO_4_ and other reagents were purchased from Sinopharm Chemical Reagent Co., Ltd. (Shanghai, China).

### 3.2. Preparation of SJP

The process of polysaccharides extraction from Semen Juglandis was performed in a thermostat-controlled water-bath apparatus (JY96, Xinzhi Bio-technology and science Inc., Ningbo, Zhejiang Province, China). Firstly, the dried raw materials were ground in a sample mill to pass through No. 60 mesh. The powdered sample was refluxed in 80% ethanol for 6 h to remove some colored materials, monosaccharides, oligosaccharides, and small molecule materials. The residue was dried at room temperature for 24 h prior to extraction. Subsequently, the residue was blended with distilled water (ratio of solution to solid, 20 to 40 *v*/*w*) at certain temperature (75–95 °C) for 90–150 min in the water bath. After incubation, the mixtures were centrifuged at 6000 rpm and the residue was re-extracted under the same condition. The combined filtrate was then concentrated by a rotary evaporator and followed by precipitation with three volumes of dehydrated ethanol. After centrifugation, the obtained crude extract was re-dissolved into distilled water and subjected to the Sevag method at least four times to remove free proteins. The resulting solution was dialyzed in regenerated cellulose tubing (*M*_w_ cut-off: 6000) against tap water for 3 days and distilled water for 2 days and then lyophilized to yield crude polysaccharide (SJP). The sediment was dissolved in distilled water to certain volume in which the SJP concentration was determined according to the classical method of Dubois et al. [29] using glucose solution as a standard reference.

### 3.3. Experimental Design of RSM for Extraction Process

Different ranges of extracting process parameters, such as extraction temperature, ratio of solution to solid, extraction time, etc., were initially studied using single factor experiments in order to get optimal central values for SJP extraction. In each single factor experiment, one factor was changed with the other factors remaining constant.

A Box-Behnken design (BBD) (Design Expert Software, trial version 7.1.3; Stat-Ease Inc., Minneapolis, MN, USA) was used to determine the best combination of extraction variables for production of SJP from Semen Juglandis. X_1_ (extraction temperature), X_2_ (extraction time), and X_3_ (ratio of solution to solid) were selected as the major variables, and the proper ranges of these 3 variables were established on the basis of “one-factor-at-a-time” trials for SJP extraction (Table 3). The design included 17 experimental trials (Table 1). A total of 5 replicates at the centre of the design were used to allow for estimation of a pure error sum of squares. Each experiment was performed in triplicate and the yield of SJP (%) was interpreted as the response (*Y*). For statistical calculations, the experimental variable xi was coded as *X_i_*, according to the following transformation equation:

(2) $$X_{i}=\frac{x_{i}-x_{0}}{\Delta x}i=1,2,3$$

where *X_i_* is the dimensionless coded value of the variable, *x*_0_ the value of *x_i_* at the center point, and Δ*x* is the step change.

Experimental data were used in an empirical 2nd order polynomial model with regression analysis. The mathematical model can be expressed as follows:

(3) $$Y=\beta_{0}+\sum_{i=1}^{3}\beta_{i}X_{i}+\sum_{i=1}^{3}\beta_{ii}X_{i}^{2}+\sum_{i=1}^{2}\sum_{j=i+1}^{3}\beta_{ij}X_{i}X_{j}$$

where *Y* is the measured response associated with each factor level combination, β_0_ is an intercept, β*_i_* represents the regression coefficients computed from the observed experimental values of *Y*, and *X_i_* represents the coded levels of independent variables [30]. The terms *X_i_X_j_* and *X_i_*^2^ represent the interaction and quadratic terms, respectively.

### 3.4. Fractionation of SJP Extract

The crude SJP obtained from the extraction of Semen Juglandis was dissolved in distilled water and sequentially loaded on a DEAE-52 column (2.0 cm × 35 cm, Whatman Co., Maidstone, Britain) with gradient elution, as detected by the phenol-sulfuric acid assay. After the column was equilibrated with distilled water, it was washed with a range of 0.05 to 1.5 mg/mL NaCl containing 0.05 M PBS at a flow rate of 1.0 mL/min, with 3 mL fraction collected. The carbohydrate was measured by a phenol-sulphuric acid method using glucose as the standard [29]. Then the major peak was pooled, dialyzed completely and finally lyophilized. The major fraction (78.3%) was then pooled and further purified by gel chromatography with a Sephacryl S-100 HR column (1.5 cm × 60 cm, Pharmacia Co., Stockholm, Sweden), which was eluted by a 0.1 mg/mL NaCl at a flow rate of 0.5 mL/min. The process was repeated several times until it presented one symmetrical peak with a purity of >99.3% in the purified SJP. Herein, we came to a conclusion that there was none of protein existed in the purified SJP, verified furtherly by no absorbance peak at 280 nm in UV spectrum. Finally, the main fraction was obtained as purified SJP after desalt and lyophilization for further study.

### 3.5. Component Analysis of the Purified SJP

The monosaccharide components of the purified SJP were analyzed by reverse-phase HPLC according to PMP (1-phenyl-3-methyl-5-pyrazolone) derivatization procedures with some modification [31]. Briefly, 11 standard monosaccharides or hydrolyzed sample were dissolved in 0.3 M NaOH (75 μL) and a 0.5 mg/mL PMP (50 μL) before the derivatization. Then the mixture was neutralized by 75 μL of 0.3 mg/mL HCl solution and was finally filtered through 0.22 μm membrane (Millipore, MA, USA). 10 μL of the resulting solution was injected into the RP-C18 column. The wavelength for UV detection was 245 nm. Elution was carried out at a flow rate of 1.0 mL/min at 25 °C. The mobile phase was a mixture of 0.05 mg/mL KH_2_PO_4_ (pH 10)–acetonitrile (83:17). Sugar identification was done by comparison with 11 reference sugars (rhamnose, ribose, arabinose, xylose, mannose, galactose, glucose, fucose, galacturonic acid, gluronic acid and glucosamine).

### 3.6. Determination of Molecular Weight

Molecular weight of the purified SJP was measured by means of Size Exclusion Chromatography with a multi-angle laser light scattering system (SEC-MALLS). The system included a pump (S-1500, SSI Technologies, Inc., Madison Heights, MI, USA), a degasser (GASTORR TG-14, GenTech Scientific Inc., Arcade, NY, USA), an injection valve (High-Pressure Injection system, Wyatt Technology, Santa Barbara, CA, USA) fitted with a 100 μL loop, SEC columns (TSK G3000 PWXL, TOSOH, Tokyo, Japan), a multi-angle laser light scattering detector (DAWN HELEOS II, Wyatt Technology, Santa Barbara, CA, USA) (λ_0_ = 658 nm), and a refractive index detector (RID-10A, SHIMADIU corporation, Kyoto, Japan). Samples were dissolved directly in ultrapure water (1–3 mg/mL), and filtered through 0.22 μm filter membranes (Millipore) prior to injection into the SEC-MALLS system. Nitrate buffer was used as the mobile phase, which containing 0.1 M NaCl containing 0.02% NaN_3_, then filtered over a filter membrane with pore size 0.22 μm, and degassed by ultrasonic cleaner for several minutes [32].

### 3.7. In Vitro Antioxidant Activities Assays

#### 3.7.1. DPPH Scavenging Assay

The DPPH scavenging abilities of three samples were determined by the method of Qu et al. [19] with some modifications. Briefly, 1 mL of SJP extract, with different concentrations, was entirely mixed with 1 mL of 0.002 mg/mL DPPH solution (use it right after it was ready). Then, the mixtures were incubated at room temperature in the dark for half an hour, and the discolorations were measured at 517 nm. Ascorbic acid as a standard was used to be compared with the samples. Triplicate measurements were carried out for each sample. The inhibition percentage of DPPH scavenging capability was calculated as the following equation:

Scavenging rate (%) = (1 − *A_s_*/*A*_0_) × 100 (4)

where *A_s_* was the absorbance of sample, and *A*_0_ was the blank control solution without the sample.

#### 3.7.2. Reducing Power Assay

The ferrous ion reducing power was measured using the previous method proposed by Sun et al. [20]. Various concentrations of SJP extract were put in the test tube, then 2.5 mL of phosphate buffer (pH 6.6, 0.2 mg/mL) and 2.5 mL of potassium ferricyanide (1%, *w*/*v*) was added and mixed thoroughly with the sample. After incubation in water bath set at 50 °C for 20 min, 2.5 mL of trichloroacetic acid (10%, *w*/*v*) was added to the reaction solution to end the reaction. And following centrifugation (1200 rpm, 10 min), 2.5 mL of the supernatant was transfered into a tube and mixed with 2.5 mL of deionized water and 0.5 mL of FeCl_3_ (0.1%, *w*/*v*). The mixture is expected to measure the reducing power though the absorbance at 700 nm after plunged into darkness at room temperature for 10 min. All the process was conducted for three times. The ascorbic acid was used as a positive control for evaluating the reducing power.

#### 3.7.3. ABTS Scavenging Assay

The assay of ABTS scavenging activity of SJP extract was quantified by the method of Wang et al. [11] with a slight modification. Dissolved in distilled water at a final concentration of 0.007 mg/mL, the ABTS was mixed with a potassium persulphate solution (0.00245 mg/mL). The reaction mixture was left at room temperature for 12 h in the dark before use. For this assay, freshly prepared ABTS^•+^ solution was diluted with ethanol (75%) to adjust its absorbance to 0.70 ± 0.02 at 734 nm wavelength. 0.1 mL of various concentrations of the sample was added into 3.9 mL of ABTS^•+^ solution, and incubating at room temperature for 10 min. Finally, the absorbance was measured at 734 nm. Ascorbic acid was used as the reference compound for measuring the ABTS scavenging activity. All the measurements were carried out in triplicate. The scavenging effect of ABTS free radical was defined as:

Scavenging effect (%) = (1 − *A_s_*/*A*_0_) × 100 (5)

where *A_s_* was the absorbance of sample and *A_0_* was the blank control solution without the sample.

#### 3.7.4. Hydroxyl Radical Scavenging Assay

The radical scavenging capability was measured using the method developed by Mao et al. [33]. 2.0 mL of FeSO_4_ (0.006 mg/mL) solution was transferred by pipette into 2.0 mL of the sample, then 2.0 mL H_2_O_2_ (0.006 mg/mL) was taken in the mixture to start the reaction. After shaken, the mixture was allowed to stay still at 37 °C for 30 min. Then 2.0 mL of salicylic acid (0.006 mg/mL) was added and mixed, standing at same temperature for another 10 min. The absorbance was measured at 510 nm. Ascorbic acid was used for comparison. All experiments were performed three times, and the capability of hydroxyl radical scavenging activity was calculated using the following equation:

Scavenging ability (%) = [1 − (*A_s_* − *A_j_*)/*A*_0_] × 100 (6)

where *A*_0_ was the absorbance control (water instead of sample solution), *A_s_* was the absorbance in the presence of the sample, and *A_j_* was the absorbance of the blank reagent (water instead of H_2_O_2_).

### 3.8. Statistical Analysis

Data were expressed as means standard error (SE) of three replicated determinations. The multiple regression analysis and analysis of variance (ANOVA) were carried out using a software Design-Expert 7.1.3 Trial to fit quadratic polynomial equations. The quality of the fitted model was expressed by the coefficient determination *R*^2^, and its statistical significant was checked by *F*-test and *p*-value.

## 4. Conclusions

It was valuable for the preparation of water-soluble polysaccharide (SJP) from the raw materials of Semen Juglandis by using response surface methodology and regression analysis. All coefficients had a significantly positive influence on the response values, regardless of the interaction between extraction temperature and ratio of solution to solid (*p* < 0.7886). The optimal conditions for the SJP extraction were as following: extraction temperature 88 °C, extraction time 125 min and ratio of solution to solid 31 mL/g. Under these conditions, experimental extraction yield of SJP was (5.73 ± 0.014)% (*n* = 5), similar to the predicted yield of 5.78%. Furtherly, the major purified fraction obtained from the crude SJP by DEAE-52 and Sephacryl S-100 chromatography was analyzed to be composed of rhamnose, galacturonic acid, galactose, arabinose and fucose in the molar ratio of 1:6.34:1.38:3.21:1.56. And the weight-average molecular weight and radius of gyration of the purified SJP in 0.1 mg/mL NaCl were determined by SEC-MALLS to be 2.76 × 10^4^ g/mol and 122 nm, respectively. More importantly, the purified SJP showed strong DPPH radical scavenging activity, reducing power, ABTS radical scavenging activity and hydroxyl radical scavenging activity. This study has provided essential information for the extraction of polysaccharides from Semen Juglandis which is expected to be used as functional health products or antioxidant.

## Figures and Tables

**Table 1:** The BBD matrix and response values for the extraction yield of SJP.

Run Order	Coded Levels			Yield of SJP (%)	
	X_1_	X_2_	X_3_	Observed	Predicted
1	−1	−1	0	3.69	3.56
2	1	−1	0	5.25	5.16
3	−1	1	0	4.99	5.09
4	1	1	0	4.92	5.05
5	−1	0	−1	4.22	4.07
6	1	0	−1	5.09	4.91
7	−1	0	1	4.38	4.56
8	1	0	1	5.13	5.28
9	0	−1	−1	3.49	3.76
10	0	1	−1	5.16	5.22
11	0	−1	1	4.98	4.93
12	0	1	1	5.17	4.90
13	0	0	0	5.69	5.67
14	0	0	0	5.69	5.67
15	0	0	0	5.68	5.67
16	0	0	0	5.65	5.67
17	0	0	0	5.64	5.67

**Table 2:** Analysis of variance (ANOVA) for the fitted quadratic polynomial model of SJP extraction.

Source	Sum of Squares	Degree of Freedom	Mean Square	*F*-Value	*p*-Value
Model	7.11	9	0.79	17.46	<0.0005
*X*_1_	1.22	1	1.22	26.85	0.0013
*X*_2_	1.01	1	1.01	22.25	0.0022
*X*_3_	0.36	1	0.36	7.95	0.0258
*X*_1_*X*_2_	0.67	1	0.67	14.88	0.0062
*X*_1_*X*_3_	0.00352	1	0.00352	0.078	0.7886
*X*_2_*X*_3_	0.55	1	0.55	12.19	0.0101
*X*_1_^2^	0.97	1	0.97	21.33	0.0024
*X*_2_^2^	0.98	1	0.98	21.59	0.0024
*X*_3_^2^	1.01	1	1.01	22.30	0.0022
Residual	0.32	7	0.045	26.85	0.0013
Lack of fit	0.31	3	0.10	137.46	0.0002
Pure error	0.003044	4	0.000761		
Cor. Total	7.43	16			
*R*^2^ = 0.9573, *R*^2^*_adj_* = 0.9025, C.V.% = 4.26%					

**Table 3:** Independent variables and their levels for Box-Behnken design.

Independent Variables	Variable Levels		
	−1	0	1
Extraction temperature (°C)	75	85	95
Extraction time (min)	90	120	150
Ratio of solution to solid (mL/g)	20	30	40

## Abbreviations

SJP	Polysaccharides from Semen Juglandis
RSM	Response surface methodology
BBD	Box-Behnken design
ANOVA	Analysis of variance
SEC-MALLS	Size Exclusion Chromatography with a multi-angle laser light scattering system

## References

[1] Verma R.S., Padalia R.C., Chauhan A., Thul S.T. (2013). Phytochemical analysis of the leaf volatile oil of walnut tree (*Juglans regia* L.) from western Himalaya. Ind. Crops Prod..

[2] Xu F., Deng G., Cheng S.Y., Zhang W.W., Huang X.H., Li L.L., Cheng H., Rong X.F., Li J.B. (2012). Molecular cloning, characterization and expression of the phenylalanine ammonia-lyase gene from *Juglans regia*. Molecules.

[3] Anderson K.J., Teuber S.S., Gobeille A., Cremin P., Waterhouse A.L., Steinberg F.M. (2001). Walnut polyphenolics inhibit in vitro human plasma and LDL oxidation. J. Nutr..

[4] Samaranayaka A.G., John J.A., Shahidi F. (2008). Antioxidant activity of English walnut (*Juglans regia* L.). J. Food Lipids.

[5] Sharma N., Ghosh P., Sharma U.K., Sinha A.K. (2012). Antioxidant potential of *Juglans regia* bark: Quantification of seven phenolic compounds by RP-HPLC. Chem. Nat. Compd..

[6] Arranz S., Pérez-Jiménez J., Saura-Calixto F. (2008). Antioxidant capacity of walnut (*Juglans regia* L.): Contribution of oil and defatted matter. Eur. Food Res. Technol..

[7] Fenoradosoa T.A., Delattre C., Laroche C., Wadouachi A., Dulong V., Picton L., Andriamadio P., Michaud P. (2009). Highly sulphated galactan from *Halymenia durvillei* (Halymeniales, Rhodophyta), a red seaweed of Madagascar marine coasts. Int. J. Biol. Macromol..

[8] Delattre C., Fenoradosoa A., Michaud P. (2011). Galactans: An overview of their most important sourcing and applications as natural polysaccharides. Braz. Arch. Biol. Technol..

[9] Delattre C., Pierre G., Gardarin C., Traikia M., Elboutachfaiti R., Isogai A., Michaud P. (2015). Antioxidant activities of a polyglucuronic acid sodium salt obtainedfrom TEMPO-mediated oxidation of xanthan. Carbohydr. Polym..

[10] Redouan E., Emmanuel P., Michelle P., Bernard C., Josiane C., Cédric D. (2011). Evaluation of antioxidant capacity of ulvan-like polymer obtained by regioselective oxidation of gellan exopolysaccharide. Food Chem..

[11] Wang J.L., Yang W., Tang Y.Y., Xu Q., Huang S.L., Yao J., Zhang J., Lei Z.Q. (2016). Regioselective sulfation of Artemisia sphaerocephala polysaccharide: Solution conformation and antioxidant activities in vitro. Carbohydr. Polym..

[12] Zhang Y.Y., Li S., Wang X.H., Zhang L.N., Cheung P.C.K. (2011). Advances in lentinan: Isolation, structure, chain conformation and bioactivities. Food Hydrocoll..

[13] Miyajima T., Ogawa H., Koike I. (2001). Water-extractable polysaccharides in marine sediments: Abundance, molecular size distribution, and monosaccharide composition. Geochim. Cosmochim. Acta.

[14] Dong C.H., Xie X.Q., Wang X.L., Zhan Y., Yao Y.J. (2009). Application of Box-Behnken design in optimisation for polysaccharides extraction from cultured mycelium of *Cordyceps sinensis*. Food Bioprod. Process..

[15] Li P.Q., Lu S.Q., Shan T.J., Mou Y., Li Y., Sun W.B., Zhou L.G. (2012). Extraction optimization of water-extracted mycelial polysaccharide from endophytic fungus *Fusarium oxysporum* Dzf17 by Response Surface Methodology. Int. J. Mol. Sci..

[16] Vitali R. (2000). Response Surface Methods for High Dimensional Structural Design Problems. Ph.D. Thesis.

[17] Liang R.J. (2008). Optimization of extraction process of *Glycyrrhiza glabra* polysaccharides by response surface methodology. Carbohydr. Polym..

[18] Qiao D.L., Hu B., Gan D., Sun Y., Ye H., Zeng X.X. (2009). Fraction optimized by using response surface methodology, purification and preliminary characterization of polysaccharides from *Hyriopsis cuminagii*. Carbohydr. Polym..

[19] Qu Y., Li C.X., Zhang C., Zeng R., Fu C.M. (2016). Optimization of infrared-assisted extraction of *Bletilla striata* polysaccharides based on response surface methodology and their antioxidant activities. Carbohydr. Polym..

[20] Sun Y.X., Liu J.C., Kennedy J.F. (2010). Extraction optimization of antioxidant polysaccharides from the fruiting bodies of *Chroogomphis rutilus* (Schaeff.: Fr.) O.K. Miller by Box-Behnken statistical design. Carbohydr. Polym..

[21] Yin G.H., Dan Y.L. (2008). Optimization of extraction technology of the *Lycium barbarum* polysaccharides by Box-Behnken statistical design. Carbohydr. Polym..

[22] Guo Q.B., Cui S., Kang J., Ding H.H., Wang Q., Wang C. (2015). Non-starch polysaccharides from American ginseng: Physicochemical investigation and structural characterization. Food Hydrocoll..

[23] Shakhmatova S.G., Toukachb P.V., Michailowac E., Makarovaa E. (2014). Structural studies of arabinan-rich pectic polysaccharides from *Abies sibirica* L. Biological activity of pectins of *A. sibirica*. Carbohydr. Polym..

[24] Wang J.T., Xu X.J., Zheng H., Li J.L., Chao D., Xu Z.H., Chen J.H. (2009). Structural characterization, chain conformation, and morphology of a β-(1→3)-d-Glucan isolated from the fruiting body of *Dictyophora indusiata*. J. Agri. Food Chem..

[25] Oliveira I., Sousa A., Ferreira I.C.F.R., Bento A., Estevinho L., Pereira J.A. (2008). Total phenols, antioxidant potential and antimicrobial activity of walnut (*Juglans regia* L.) green husks. Food Chem. Toxicol..

[26] Luo A.X., He X.J., Zhou S.D., Fan Y.J., Luo A.S., Chun Z. (2010). Purification, composition analysis and antioxidant activity of the polysaccharides from *Dendrobium nobile* Lindl. Carbohydr Polym..

[27] Yu H.H., Liu X.G., Xing R.E., Liu S., Guo Z.Y., Wang P.B., Li C.P., Li P.C. (2006). In vitro determination of antioxidant activity of proteins from jellyfish *Rhopilema esculentum*. Food Chem..

[28] Yang X.M., Yu W., Ou Z.P., Ma H.L., Liu W.M., Ji X.L. (2009). Antioxidant and immunity activity of water extract and crude polysaccharide from *Ficus carica* L. fruit. Plant Food. Hum. Nutr..

[29] Dubois M., Gilles K.A., Hamilton J.K., Rebers P.A., Smith F. (1956). Colorimetric method for determination of sugars and related substances. Anal. Chem..

[30] Ren X.Y., He L., Cheng J.W., Chang J.M. (2014). Optimization of the solid-state fermentation and properties of a polysaccharide from *Paecilomyces cicadae* (Miquel) Samson and its antioxidant activities in vitro. PLoS ONE.

[31] He L., Ji P.F., Gong X.G., Li W.Q., Cheng J.W., Qian H., Song X.L. (2016). Physico-chemical characterization, antioxidant and anticancer activities in vitro of a novel polysaccharide *Melia toosendan* Sieb. Et Zucc fruit. Int. J. Biol. Macromol..

[32] Lv Y., Yang X.B., Zhao Y., Ruan Y., Yang Y., Wang Z.Z. (2009). Separation and quantification of component monosaccharides of the tea polysaccharides from *Gynostemma pentaphyllum* by HPLC with indirect UV detection. Food Chem..

[33] Mao G., Zou Y., Feng W., Wang W., Zhao T., Ye C., Zhu Y., Wu X.S., Yang L.Q., Wu X.Y. (2014). Extraction, preliminary characterization and antioxidant activity of Se-enriched Maitake polysaccharide. Carbohydr. Polym..

